# 6‐Shogaol Attenuates Doxorubicin‐Induced Cardiac and Skeletal Muscle Atrophy by Inhibiting E3 Ubiquitin Ligases and Necroptosis

**DOI:** 10.1002/ptr.70276

**Published:** 2026-03-16

**Authors:** Xipeng Sun, Yaxian Wang, Quanjun Yang, Bo Xin, Jinlu Huang, Cheng Guo

**Affiliations:** ^1^ Department of Pharmacy Shanghai Sixth People's Hospital Affiliated to Shanghai Jiao Tong University School of Medicine Shanghai China; ^2^ School of Pharmacy Shanghai University of Traditional Chinese Medicine Shanghai China

**Keywords:** 6‐shogaol, cardiac atrophy, doxorubicin, E3 ubiquitin ligases, necroptosis, skeletal muscle atrophy

## Abstract

Doxorubicin (DOX) is an effective anticancer agent, but it not only induces dose‐dependent cardiotoxicity but also causes severe cardiac and skeletal muscle atrophy. Recent studies have indicated that cardiac atrophy may play an important role in DOX‐induced cardiotoxicity. However, the mechanisms responsible for DOX‐induced cardiac and skeletal muscle atrophy remain unclear. 6‐Shogaol (6‐SH) is a bioactive component from ginger, which exhibits various bioactive effects and can alleviate cisplatin‐induced cachexia. In this work, we systematically assessed the effects of DOX and/or 6‐SH on heart and skeletal muscle, as well as the underlying mechanisms. C57BL/6 mice were treated with DOX (5 mg/kg/3d, 4 doses, intraperitoneal) and given high or low doses of 6‐SH (10/2.5 mg/kg, qd, intraperitoneal) for 14 days. Body weight, skeletal muscle mass, heart mass, grip strength, food intake and cardiac function were significantly reduced in DOX‐treated mice, with these impairments notably ameliorated by 6‐SH. Unexpectedly, 6‐SH synergistically enhanced the antitumor efficacy of DOX. The DOX‐induced significant decline in mitochondrial levels and slow‐to‐fast fiber type shift in skeletal muscle were notably attenuated by 6‐SH treatment. Meanwhile, 6‐SH exerted a protective effect against DOX‐induced increases in cardiac oxidative stress, cardiac injury markers and inflammatory cytokines. DOX significantly upregulated the E3 ubiquitin ligases Atrogin1 and MuRF1 and downregulated MyoD and MyoG in the heart and skeletal muscle. Furthermore, DOX activated necroptosis, as evidenced by increased phosphorylation of receptor‐interacting protein kinase (RIPK) 1, RIPK3, and mixed‐lineage kinase domain‐like (MLKL). 6‐SH negatively regulated E3 ubiquitin ligases and necroptosis, while upregulating myogenic regulatory factors. In conclusion, 6‐SH attenuated DOX‐induced cardiac atrophy, skeletal muscle atrophy and cardiotoxicity by inhibiting E3 ubiquitin ligases and necroptosis.

## Graphical abstract

Doxorubicin induces cardiac and skeletal muscle atrophy by upregulating E3 ubiquitin ligases, inhibiting myogenic regulatory factors, and activating necroptosis. Cardiac atrophy can further exacerbate cardiotoxicity. 6‐Shogaol negatively regulates these processes and attenuates doxorubicin‐induced cardiac and skeletal muscle atrophy.

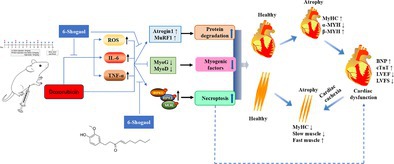

## 1 Introduction

Doxorubicin (DOX), an anthracycline antineoplastic, has been widely used as a chemotherapeutic treatment for various malignancies because of its broad spectrum of antitumor activity (Gianni et al. 2008). However, its clinical use is limited by dose‐dependent cardiotoxicity, which is characterized by irreversible dilated cardiomyopathy and heart failure, particularly in long‐term survivors of cancer (Gianni et al. 2008; Rawat et al. 2021). The pathophysiological mechanisms underlying DOX‐induced cardiotoxicity are complex and multifactorial, which involve the generation of reactive oxygen species (ROS), mitochondrial dysfunction, programmed cell death, and inflammation (Christidi and Brunham 2021; Rawat et al. 2021; Wu et al. 2022). Additionally, recent studies have accentuated the critical role of cardiac atrophy in DOX‐induced cardiotoxicity (Chen et al. 2022; Willis et al. 2019, 2009). Cardiac atrophy, which is characterized by a reduction in myocardial cell size and heart mass, is increasingly recognized as a significant contributor to the progressive decline in cardiac function observed in patients treated with DOX (Hiensch et al. 2020; Willis et al. 2019). This form of cardiac remodeling is distinct from the pathological hypertrophy often associated with hypertension and other cardiovascular diseases, where the cardiac muscle increases in size but not functionally (Chen et al. 2022). The E3 ubiquitin ligases Atrogin1 and MuRF1, which are muscle‐specific regulators, have been identified as key mediators of cardiac atrophy following DOX exposure (Willis et al. 2019; Yamamoto et al. 2008). These findings are particularly significant, as they suggest that therapies targeting Atrogin1 and MuRF1 could mitigate the cardiotoxicity of DOX, preserving cardiac function in cancer patients undergoing chemotherapy.

In addition, DOX may directly induce acute or chronic toxicity in skeletal muscle, leading to muscle weakness, atrophy, and fatigue, as well as the development of cancer cachexia (Hiensch et al. 2020; Kavazis et al. 2014). Skeletal muscle atrophy, characterized by a decrease in muscle mass and strength, is associated with significant morbidity and reduced quality of life (Shachar et al. 2016). The molecular pathways and mechanisms include mitochondrial dysfunction, increased production of ROS, activation of proteolytic systems, the ubiquitin–proteasome pathway and the autophagy‐lysosomal pathway (Hiensch et al. 2020; Rausch et al. 2021). The toxicity of DOX to cardiac and skeletal muscle may significantly contribute to cancer cachexia, mortality, and decreased quality of life. Understanding the details and differences of DOX‐induced cardiac and skeletal muscle atrophy is important for the discovery of novel and effective therapeutic approaches.

Necroptosis is an important form of programmed cell death that has been identified in various inflammatory and degenerative diseases. The receptor‐interacting protein kinase (RIPK) signaling pathway, which mainly includes RIPK1 and RIPK3, is a critical regulator of necroptosis (He et al. 2009; Newton 2015; Silke et al. 2015). RIPK1 can either promote cell survival or induce cell death, depending on its activation state and interaction with other signaling molecules (Martens et al. 2020; Newton et al. 2014). RIPK3 is a key mediator of necroptosis, which occurs following the formation of a necrosome complex with RIPK1(Kaiser et al. 2013; Newton et al. 2014). Once RIPK1 and RIPK3 are phosphorylated, mixed‐lineage kinase domain‐like (MLKL) is activated, leading to MLKL oligomerization and translocation to the cell membrane, subsequently causing membrane pore formation and rupturing (Martens et al. 2020; Newton 2015). Studies have shown that DOX can trigger the phosphorylation and activation of RIPK1 and RIPK3, leading to myocardial necroptosis and cardiac dysfunction (Khuanjing et al. 2021; Yu et al. 2020). However, the role of necroptosis in cardiac and skeletal muscle atrophy induced by tumor or chemotherapy has rarely been reported. Interestingly, necroptosis is involved in muscle development and regeneration (Qaisar 2024). Guo et al. were the first and only group to demonstrate that necroptosis mediates muscle protein degradation in lipopolysaccharide‐induced cachexia (Guo et al. 2023). Therefore, investigating the role of necroptosis in DOX‐induced cardiac and skeletal muscle atrophy is necessary.

**FIGURE 1:**
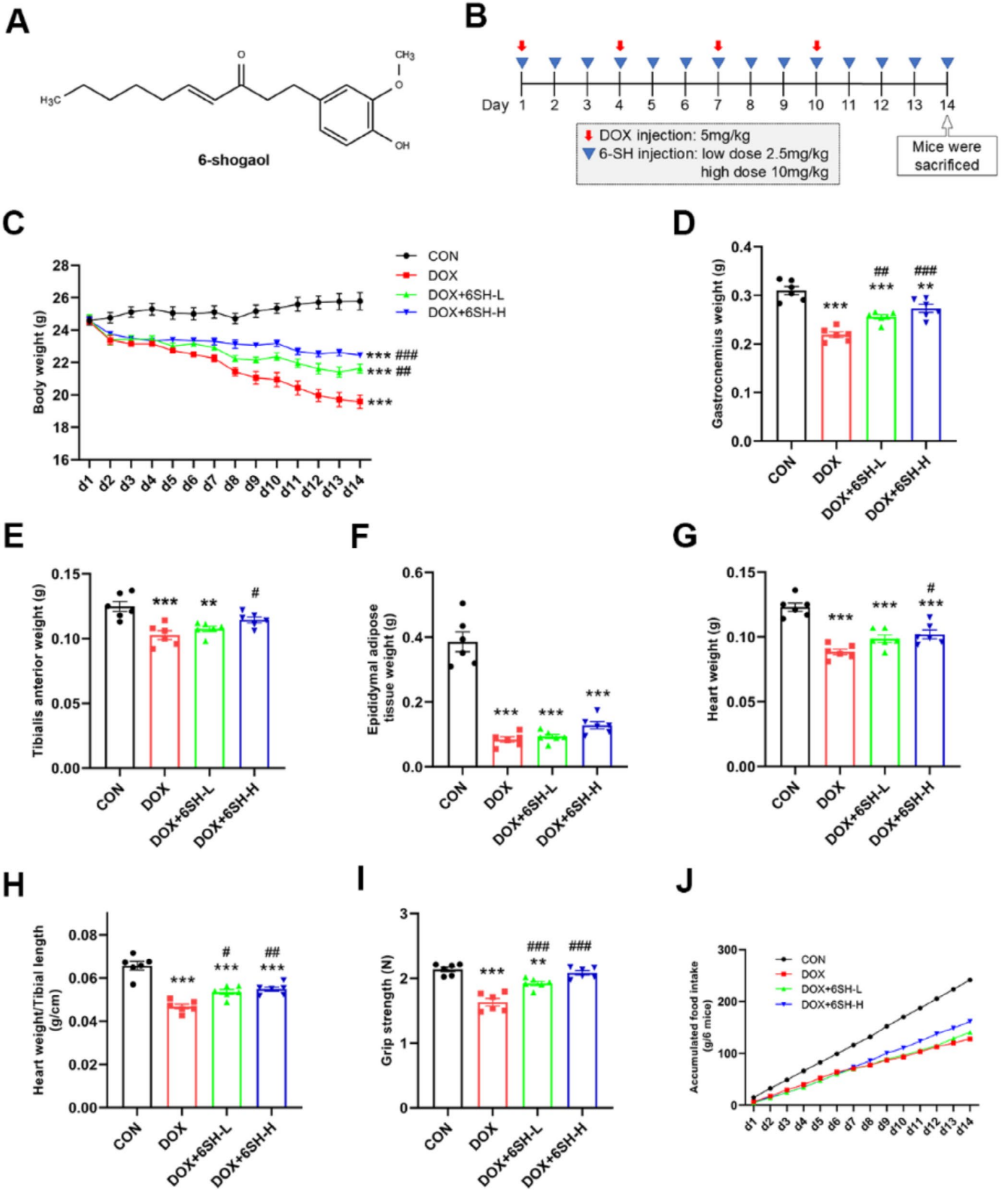
6‐SH alleviated the loss of body weight, muscle weight, heart weight, grip strength and food intake in healthy mice after DOX treatment. (A) The chemical structure of 6‐SH. (B) Schematic representation of the experimental protocol. C57BL/6 mice were treated with DOX (5 mg/kg/3d, 4 doses, i.p.) and given low doses of 6‐SH (2.5 mg/kg, qd, i.p.) or high doses of 6‐SH (10 mg/kg, qd, i.p.) at the same time for 14 days. (C) Body weight was measured every day. (D–I). The gastrocnemius, tibialis anterior, epididymal adipose tissue and heart were weighed, and the heart weight‐to‐tibial length ratio and grip strength were measured. (J) The cumulative food intake of each group, *n* = 6/group. The data are shown as the means ± SEM, *n* = 6/group, **p < 0.01, ***p < 0.001 versus the CON group; #*p* < 0.05, ##*p* < 0.01, ###*p* < 0.001 versus the DOX group.

6‐Shogaol (6‐SH) is one of the major bioactive components of ginger ( *Zingiber officinale* ) (Figure 1A). 6‐SH has strong antioxidant, anti‐inflammatory, antimicrobial, and anticancer properties; thus, it holds therapeutic potential for atherosclerosis, heart failure, bacterial and fungal infections, gastric ulcers, and vomiting (Bischoff‐Kont and Furst 2021; Figueroa‐Gonzalez et al. 2024; Yang et al. 2024). 6‐SH exerts cardioprotective effects against a range of cardiovascular diseases (Kawase et al. 2023; Li et al. 2022). Our previous study revealed that 6‐SH alleviates cisplatin‐induced cachexia and skeletal muscle atrophy (Guo et al. 2021). Therefore, this study investigated the effects and underlying mechanisms of DOX on cardiac and skeletal muscle atrophy, as well as whether 6‐SH exerts a protective effect against DOX‐induced cardiac and skeletal muscle atrophy. In the present study, we evaluated DOX‐mediated alterations in heart and skeletal muscle, and the results revealed a significant reduction in the mass of both tissues following DOX exposure. We hypothesized that 6‐SH potentially regulates E3 ubiquitin ligases, myogenic factors, ROS production, inflammation and necroptosis, thereby attenuating DOX‐induced cardiac and skeletal muscle atrophy and ultimately alleviating DOX‐induced cardiotoxicity.

## 2 Materials and Methods

### 2.1 Animals

Six‐ to eight‐week‐old male C57BL/6 mice were purchased from the Laboratory Animal Business Department of Shanghai Family Planning Research Institute (Shanghai, China). The mice were housed in cages at a constant temperature and humidity with a 12 h light/dark cycle. All the mice had free access to water and a regular chow diet for adaptation to the new environment before the experiments began. All the animal experiments described in this study were performed in strict accordance with the ethical requirements of the Laboratory Animal Research Center, Shanghai Sixth People's Hospital Affiliated to Shanghai Jiao Tong University School of Medicine, and the principles of the Guide for the Care and Use of Laboratory Animals (8th edition, 2011). The experimental protocols were approved by the Animal Welfare Ethics Committee of Shanghai Sixth People's Hospital.

### 2.2 Animal Experimental Protocol

**FIGURE 2:**
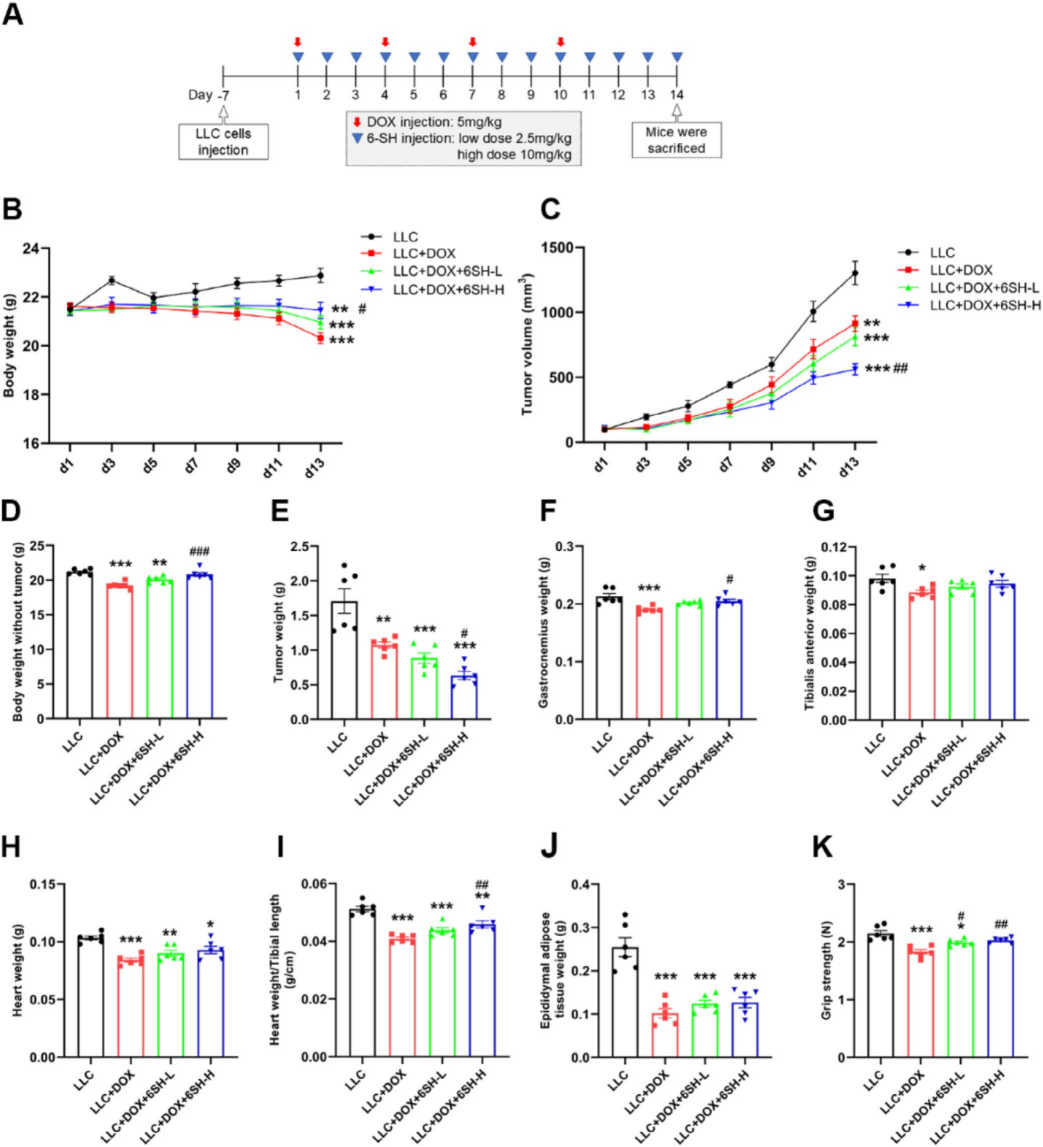
6‐SH prevented DOX‐induced cachexia symptoms and heart atrophy in LLC tumor‐bearing mice and enhanced the antitumor effect of DOX. (A) Schematic representation of the experimental protocol. C57BL/6 mice were implanted with LLC cells in the right flank, treated with DOX (5 mg/kg/3d, 4 doses, i.p.) and given low doses of 6‐SH (2.5 mg/kg, qd, i.p.) or high doses of 6‐SH (10 mg/kg, qd, i.p.) at the same time for 14 days. (B) Body weight was measured every 2 days. (C) The tumor size was assessed every 2 days after the start of drug administration. (D) After the mice were sacrificed, body weights without tumors were measured. (E–K) The gastrocnemius, tibialis anterior, epididymal adipose tissue and heart were weighed, and the heart weight‐to‐tibial length ratio and grip strength were measured. The data are shown as the means ± SEM, *n* = 6/group, *p < 0.05, **p < 0.01, ***p < 0.001 versus the CON group; #*p* < 0.05, ##*p* < 0.01, ###*p* < 0.001 versus the DOX group.

The study included two experimental protocols. For experiment 1, 24 healthy C57BL/6 male mice were randomly divided into four groups (*n* = 6/group): the normal control group (CON) (0.1 mL normal saline, intraperitoneal (i.p.); 0.1 mL 10% DMSO corn oil, i.p.), the DOX group (DOX (Shenzhen Main Luck Pharmaceuticals Inc., Shenzhen, China), 5 mg/kg/3d, i.p.; 0.1 mL 10% DMSO corn oil, i.p.), the DOX + 6SH‐L group (DOX, 5 mg/kg/3d, i.p.; 6‐SH (Chengdu Herbpurify Co. LTD., Chengdu, China), 2.5 mg/kg/d, i.p., dissolved in 10% DMSO, 90% corn oil), and the DOX + 6SH‐H group (DOX, 5 mg/kg/3d, i.p.; 6‐SH, 10 mg/kg/d, i.p., dissolved in 10% DMSO, 90% corn oil). The detailed experimental protocols are described in Figure 1B. For experiment 2, the mice were injected subcutaneously on the right side of the back with 1 × 10^6^ mouse Lewis lung cancer (LLC) cells. The LLC‐bearing C57BL/6 male mice were randomly divided into four groups (*n* = 6 per group): the LLC group (0.1 mL normal saline, i.p.; 0.1 mL 10% DMSO corn oil, i.p.), the LLC + DOX group (DOX, 5 mg/kg/3d, i.p.), the LLC + DOX + 6SH‐L group (DOX, 5 mg/kg/3d, i.p.; 6‐SH, 2.5 mg/kg/d, i.p.; dissolved in 10% DMSO, 90% corn oil), and the LLC + DOX + 6SH‐H group (DOX, 5 mg/kg/3d, i.p.; 6‐SH, 10 mg/kg/d, i.p.; dissolved in 10% DMSO, 90% corn oil). The detailed experimental protocols are described in Figure 2A. The doses of 6‐SH used in this study were chosen on the basis of our preliminary study (Guo et al. 2021). We monitored the body weight, tumor volume and food intake of the mice between 10:00 and 12:00 during the experiments. Tumor volumes were measured every 2 days and calculated according to the following formula: 0.5 × tumor length × (tumor width)^2^. The experiments were terminated after 14 days of intervention. Echocardiography and grip strength were tested at the end of the experiment. The grip strength was measured three times via a grip strength meter (SH‐20, NSCING, Nanjing, China), and the average was used for analysis. The mice were subsequently anaesthetized and sacrificed to enable collection of tissues and blood. The heart, gastrocnemius, tibialis anterior, epididymal adipose tissue and tumor were immediately excised after anaesthetization, weighed, and stored.

### 2.3 Echocardiographic Assessment

Transthoracic echocardiographic assessments were conducted on animals under mild anesthesia (2% isoflurane), ensuring a heart rate within the range of 300–400 beats per minute; a standardized procedure was followed. Echocardiographic parameters, including parasternal long‐axis and short‐axis views, were captured via a high‐frequency ultrasound probe with a transthoracic echocardiography system (Vevo 2100, FUJIFILM VisualSonics Inc., Toronto, ON, Canada) in B‐Mode. M‐Mode was used to evaluate cardiovascular function in the long/short‐axis direction, as were parameters such as wall thickness, volume, and ejection fraction during the cardiac cycle. Doppler mode was employed to assess the energy status of cardiac blood flow. The following parameters were measured and calculated as indicators of cardiac systolic and diastolic function: left ventricular ejection fraction (LVEF), left ventricular fractional shortening (LVFS), left ventricular internal diameter at diastole (LVIDd), left ventricular internal diameter at systole (LVIDs), left ventricular systolic volume (LVvol;s), left ventricular diastolic volume (LVvol;d), left ventricular posterior wall thickness at systole (LVPWs), left ventricular posterior wall thickness at diastole (LVPWd), left ventricular septal thickness at diastole (LVS;d), left ventricular septal thickness at systole (LVS;s), the E/A ratio, and left ventricular mass (LV mass). The echocardiographic observers were blinded to the treatment conditions to ensure that the data were unbiased.

### 2.4 Histology Examination

Gastrocnemius, heart and epididymal adipose tissues were fixed in 4% paraformaldehyde and then embedded in paraffin. The paraffin blocks were cut into 4 μm thick slices and stained with a haematoxylin and eosin (H&E) kit (Yuxiu Biotechnology, Shanghai, China), Masson's trichrome staining kit (Solarbio, Beijing, China), and wheat germ agglutinin kit (WGA) (Yuxiu Biotechnology, Shanghai, China). H&E‐stained sections of gastrocnemius and epididymal adipose tissue were used for cross‐sectional area (CSA) analysis. The CSA of the cardiac muscle was measured on the WGA‐stained sections, and myocardial fibrosis was evaluated via Masson's trichrome staining. The CSA was analyzed with ImageJ software (NIH, MD, USA).

### 2.5 Succinate Dehydrogenase (SDH) Staining

Fresh gastrocnemius tissue was immersed in liquid nitrogen and sectioned on a cryostat. The slices were placed in SDH medium and incubated for 30 min in a 37°C incubator. After being washed with distilled water, the slices were transferred through a gradient of acetone solutions (30%, 60%, and 90%), each gradient for 1 min. After the slices were washed with distilled water, they were dehydrated via transfer through a gradient of ethanol solutions (70%, 90%, and 100%) and then mounted in a glycerine gelatine.

### 2.6 Fast and Slow Muscle‐Type Assay

Gastrocnemius tissue was fixed in 4% paraformaldehyde and sectioned. Fast‐twitch and slow‐twitch fibers in mouse skeletal muscle were stained with specific monoclonal antibodies. The sections were incubated with primary antibodies anti‐MyHC I (ab11083, Abcam, MA, USA) and anti‐MyHC II (ab51263, Abcam, MA, USA) at 4°C overnight. Subsequently, the slices were incubated at room temperature for 1 h with the corresponding secondary antibodies in phosphate‐buffered saline (PBS), along with 4′,6‐diamidino‐2‐phenylindole (DAPI) (C1006, Beyotime Biotechnology, Shanghai, China). The secondary antibodies used were anti‐mouse IgG1 Alexa Fluor 488 (green)‐labeled MyHC I antibody and anti‐mouse IgG1 Alexa Fluor 594 (red)‐labeled MyHC II antibody. The fluorescence intensity of fast‐twitch and slow‐twitch fiber markers was measured via ImageJ software.

### 2.7 Measurement of Oxidative Stress Levels

The gastrocnemius and heart tissues were precisely weighed, and then a solution of normal saline was added at a ratio of 1:9 (w/v, g/mL) to prepare a 10% tissue homogenate. This homogenate was prepared for the subsequent analysis of malondialdehyde (MDA) (A003‐2), superoxide dismutase (SOD) (A001‐3), glutathione (GSH) (A006‐1), and catalase (CAT) (A007‐1) levels. The protein concentration of the homogenate was determined via a Coomassie Brilliant Blue assay kit. All the results were measured with an enzyme labeler (BioTek, VT, USA) according to the manufacturer's instructions. All kits were purchased from Jiancheng Bioengineering Institute (Nanjing, China).

### 2.8 Enzyme‐Linked Immunosorbent Assay (ELISA)

Mouse serum tumor necrosis factor alpha (TNF‐α) (KE10002, Proteintech, Wuhan, China), interleukin 6 (IL‐6) (KE10007, Proteintech, Wuhan, China), cardiac troponin T (cTnT) (E‐EL‐M1801, Elabscience, Wuhan, China), and brain natriuretic peptide (BNP) (ELAM‐BNP, RayBio, USA) levels were quantified using ELISA kits. The assays were performed according to the manufacturer's instructions, and all the results were quantified by measuring the OD value with an enzyme labeler (BioTek, VT, USA).

### 2.9 Western Blot Analysis

Gastrocnemius and heart samples were homogenized and lysed in RIPA buffer (WB3100, NCM Biotech, Suzhou, China) containing protease inhibitor and phosphatase inhibitor (P001, P003, NCM Biotech, Suzhou, China) according to the manufacturer's instructions. The lysates were subsequently centrifuged at 12,000 × *g* for 10 min at 4°C, after which the supernatants were transferred to new centrifuge tubes. The protein concentrations were measured via a bicinchoninic acid (BCA) protein concentration assay kit (P0011, Beyotime, Shanghai, China). The proteins were added to 5× SDS–PAGE loading buffer (WB2100, NCM Biotech, Suzhou, China) and denatured by heating for 15 min at 100°C. A total of 40 μg of protein was loaded and separated via a 7.5%–12.5% sodium dodecyl sulfate–polyacrylamide gel electrophoresis (SDS–PAGE) and subsequently transferred to a polyvinylidene difluoride membrane (Millipore Corporation, Bedford, USA). After being blocked in Tris‐buffered saline (TBS) containing 5% skim milk or BSA for 1 h at room temperature, the membrane was incubated with primary antibodies at 4°C overnight and then with goat anti‐mouse or anti‐rabbit IgG HRP (7074, 7076, Cell Signaling, MA, USA) (1:10,000) for 1 h at room temperature. An enhanced chemiluminescence (ECL) kit (SQ201L, Epizyme, Shanghai, China) was used to visualize the protein bands, and the chemiluminescent signals on the membrane were detected with a Tanon 5200Multi instrument (Tanon, Shanghai, China). The following primary antibodies (1:1000) were used: anti‐MyhC (MAB4470, R&D Systems, MN, USA), anti‐MyoD (M6190, Sigma‐Aldrich, SL, USA), anti‐MyoG (ab1835, Abcam, MA, USA), anti‐Atrogin1/MAFbx (ab168372, Abcam, MA, USA), anti‐MuRF1 (ab172479, Abcam, MA, USA), anti‐MYH6/α‐MYH (A12964, ABclonal Technology, Wuhan, China), anti‐MYH7/β‐MYH (A7564, ABclonal Technology, Wuhan, China), anti‐phospho‐RIPK1/RIP (S166) (AP1230, ABclonal Technology, Wuhan, China), anti‐RIPK1/RIP (A7414, ABclonal Technology, Wuhan, China), anti‐phospho‐RIP3 (T231/S232) (AP1260, ABclonal Technology, Wuhan, China), anti‐RIPK3 (A5431, ABclonal Technology, Wuhan, China), anti‐phospho‐MLKL (Ser358) (91,689, Cell Signaling Technology, MA, USA), anti‐MLKL (14,993, Cell Signaling Technology, MA, USA), anti‐β‐Tubulin (A12289, ABclonal Technology, Wuhan, China), and anti‐GAPDH (A19056, ABclonal Technology, Wuhan, China). Tubulin and GAPDH were used as internal controls. Protein blots were quantified via ImageJ software.

### 2.10 RNA Isolation, Reverse Transcription and Real‐Time Quantitative Polymerase Chain Reaction (RT‐qPCR)

Total RNA was extracted from the gastrocnemius and heart with a FastPure Plant Total RNA Isolation Kit (RC401, Vazyme, Nanjing, China) according to the manufacturer's instructions. The total RNA content was evaluated via a Nanodrop Lite spectrophotometer (Thermo Fisher Scientific, Wilmington, DE, USA). Complementary DNA (cDNA) was synthesized from 2000 ng of total RNA with HiScript II Q Select RT SuperMix (R223‐01, Vazyme, Nanjing, China). RT–qPCR was conducted using a reaction mixture containing SYBR Green master mix (Q711‐02‐AA, Vazyme, Nanjing, China). The results were calculated using the 2^−ΔΔct^ relative quantification method and normalized to RPS18 gene levels. The sequences of the primers are listed in Table 1.

**TABLE 1:** Primers used for RT‐qPCR.

Primer	Sequence
RPS18 (forward)	GTAACCCGTTGAACCCCATT
RPS18 (reverse)	CCATCCAATCGGTAGTAGCG
Myh2 (forward)	GCGACAGACACCTCCTTCAAGAAC
Myh2 (reverse)	GTCCAGCCAGCCAGTGATGTTG
Myh4 (forward)	TGATGCAGGCTGAGATCGAGGAG
Myh4 (reverse)	TTGGTGTTGATGAGGCTGGTGTTC
Myh6 (forward)	GCAGCCCAGTACCTCCGAAAG
Myh6 (reverse)	TGTCATCAGGCACGAAGCACTC
Myh7 (forward)	GCAAGACGGTGACTGTGAAGGAG
Myh7 (reverse)	GGTTGACGGTGACGCAGAAGAG
MyoG (forward)	AGAGGAAGTCTGTGTCGGTGGAC
MyoG (reverse)	GTAGGCGCTCAATGTACTGGATGG
MyoD (forward)	CGTGGCAGCGAGCACTACAG
MyoD (reverse)	CGACACAGCCGCACTCTTCC
MurF1 (forward)	TGCCTACTTGCTCCTTGTGC
MurF1 (reverse)	CACCAGCATGGAGATGCAGT
Atrogin1 (forward)	GTCGGCAAGTCTGTGCTGGTG
Atrogin1 (reverse)	AGGCAGGTCGGTGATCGTGAG

### 2.11 Statistical Analysis

All the data are presented as the mean ± standard error of mean (SEM) and were analyzed via GraphPad Prism 8.0.2. For comparisons of two sets of measurements, a *t*‐test was performed. For comparisons of three or more groups of measurements, one‐way ANOVA followed by a post hoc comparison with Tukey's test was performed. A *p* value < 0.05 was considered to indicate statistical significance.

## 3 Results

### 3.1 6‐SH Alleviated DOX‐Induced Cachexia Symptoms and Cardiac Atrophy

To explore the effects of 6‐SH on DOX‐induced cachexia symptoms and cardiac atrophy, C57BL/6 mice were treated with DOX (5 mg/kg/3d, 4 doses, i.p.) at an amount that accumulated to 20 mg/kg, and they were simultaneously given high or low doses of 6‐SH (10/2.5 mg/kg, qd, i.p.) for 14 days (Figure 1B). DOX caused a significant decrease in body weight, but 6‐SH mitigated weight loss in a dose‐dependent manner (Figure 1C). Furthermore, 6‐SH ameliorated DOX‐induced muscle wasting in the gastrocnemius and tibialis anterior (Figure 1D,E). DOX induced severe epididymal adipose weight loss, but 6‐SH did not alleviate this effect (Figure 1F). DOX caused a decrease in heart weight and heart weight‐to‐tibial length ratio, which was partially reversed by 6‐SH treatment (Figure 1G,H). Strength loss is a hallmark of cachexia, and DOX caused a reduction in muscle grip strength; however, 6‐SH treatment enhanced the grip strength of the mice (Figure 1I). Over time, the food intake of DOX‐treated mice decreased, but a high dose of 6‐SH slowed the decrease in food intake (Figure 1J).

To further investigate the effects of 6‐SH on cachexia symptoms and cardiac atrophy associated with DOX treatment in tumor‐bearing mice, as well as to determine whether 6‐SH interferes with the antitumor efficacy of DOX LLC tumor‐bearing mice were treated with DOX and 6‐SH (Figure 2A). Body weight changes were assessed, and the results revealed that 6‐SH alleviated DOX‐induced weight loss in tumor‐bearing mice in a dose‐dependent manner (Figure 2B). Tumor size was assessed every 2 days; DOX‐treated tumors were significantly smaller than those in the LLC group. Furthermore, compared with the LLC + DOX group, the size and weight of the tumors in the LLC+DOX+6SH‐H group were markedly lower (Figure 2C,E). The changes in muscle (gastrocnemius and tibialis anterior), epididymal adipose tissue, heart weight, heart weight‐to‐tibial length ratio, and grip strength were consistent with the aforementioned findings (Figure 2F–K). These results suggest that 6‐SH may have a protective role against cachexia and cardiac atrophy in the context of DOX treatment without compromising its antitumor efficacy. To eliminate tumor‐host interactions, samples obtained from healthy mice were used for subsequent studies. This approach ensures a more accurate assessment of the long‐term toxicity of DOX and is not influenced by the tumor burden.

### 3.2 6‐SH Alleviated DOX‐Induced Skeletal Muscle Fiber Atrophy

**FIGURE 3:**
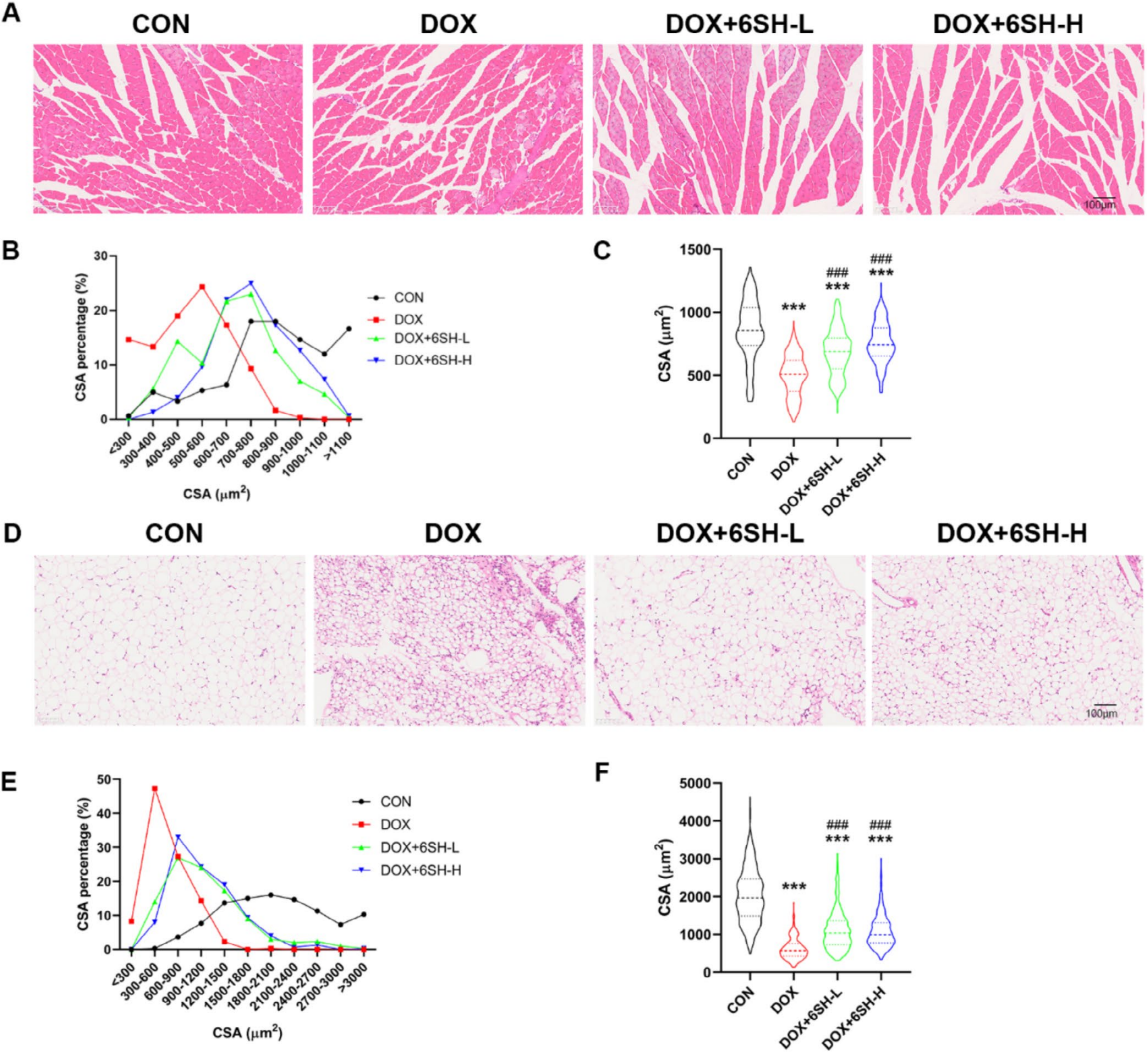
6‐SH alleviated the reduction in CSA of skeletal muscle fibers and epididymal fat induced by DOX. (A) H&E‐stained sections of mouse gastrocnemius. (B) The CSA was measured via ImageJ software, and the gastrocnemius muscle fibers CSA distribution was shown (*n* = 300). (C) Statistical analysis of gastrocnemius muscle fibers CSA (*n* = 300). (D) H&E‐stained sections of epididymal fat. (E) The epididymal fat CSA distribution was shown (*n* = 300). (F) Statistical analysis of epididymal fat CSA (*n* = 300). ***p < 0.001 versus the CON group; ###*p* < 0.001 versus the DOX group.

We evaluated the effect of 6‐SH on skeletal muscle fibers in healthy mice after DOX treatment. Atrophied muscle fibers can be identified by their reduced CSA. Following H&E staining of the muscle tissues, the CSA of the gastrocnemius muscle fibers was measured (Figure 3A). In the CON group, the CSA of skeletal muscle fibers was mainly 700–900 μm^2^, and the average CSA was 857.4 μm^2^. However, the skeletal muscle fibers in the DOX group were atrophied, and the average CSA was approximately 498.9 μm^2^. After the 6‐SH treatment, the CSAs of the skeletal muscle fibers increased significantly in both the DOX + 6SH‐L and DOX + 6SH‐H groups; additionally, the CSA distribution curve shifted rightward along the X‐axis, with the CSA values concentrated in the higher range (Figure 3B,C). During DOX‐induced chronic toxicity, epididymal fat weight decreased markedly. Although 6‐SH failed to reverse DOX‐induced epididymal fat loss, it alleviated the decrease in the CSA of epididymal fat (Figure 3D–F).

### 3.3 6‐SH Prevented DOX‐Induced Decreases in Mitochondrial Levels in the Gastrocnemius and Muscle Fiber Shift

**FIGURE 4:**
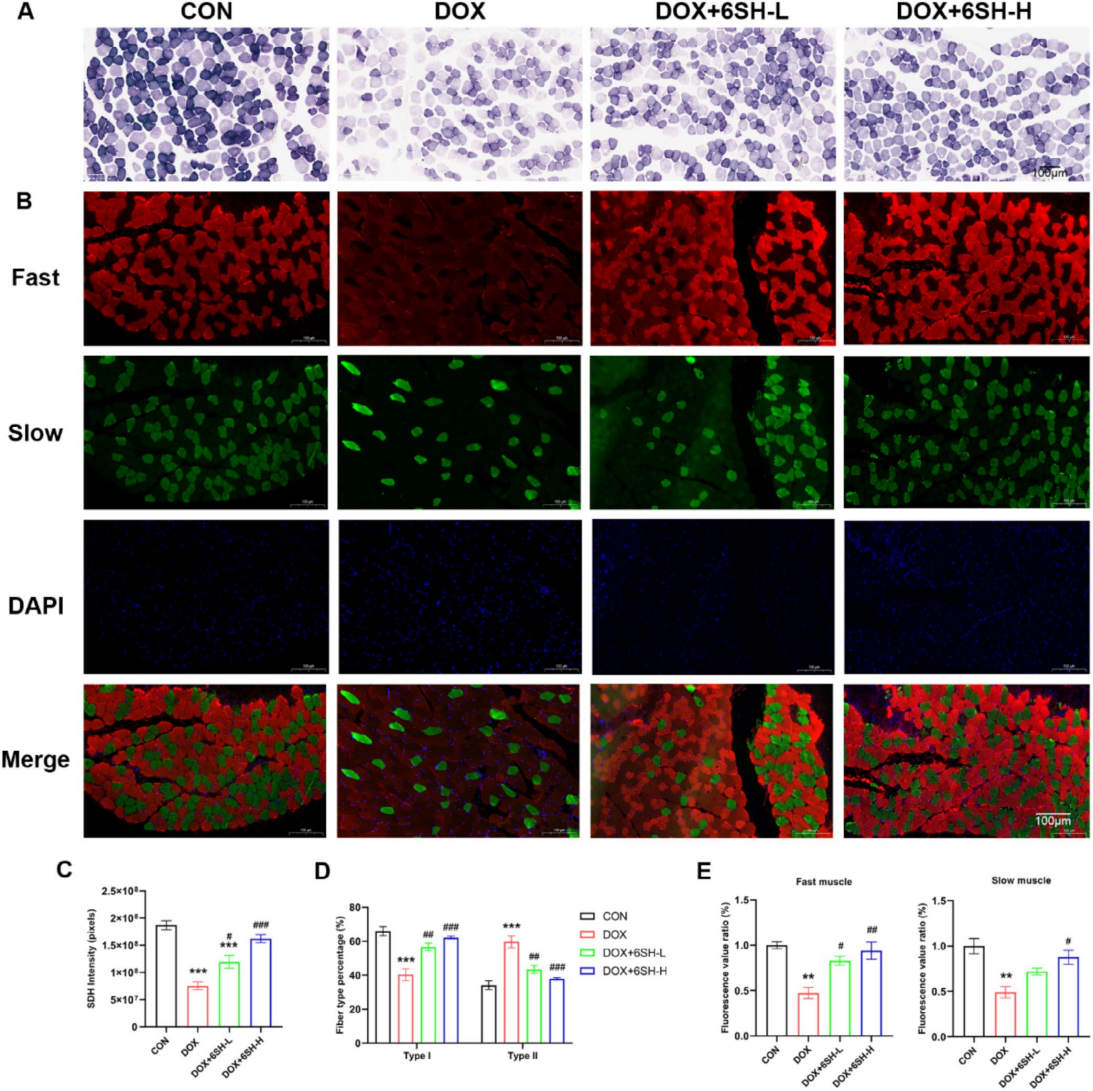
Effects of 6‐SH on SDH activity and the distribution of muscle fiber types in the gastrocnemius after DOX treatment. (A) SDH‐stained sections of mouse gastrocnemius tissue. (B) Sections of mouse gastrocnemius tissue were stained with MyHC I and MyHC II and observed under a fluorescence microscope. (C) The SDH intensity was quantitatively analyzed via ImageJ software. (D) Muscle fiber type distribution in the gastrocnemius. (E) Quantitative analysis of fluorescence intensity in fast‐twitch fibers and slow‐twitch fibers via ImageJ software. **p < 0.01, ***p < 0.001 versus the CON group; #*p* < 0.05, ##*p* < 0.01, ###*p* < 0.001 versus the DOX group.

SDH activity is positively correlated with the number of mitochondria, so we assessed the mitochondria levels in the gastrocnemius through SDH staining. As shown in Figure 4A,C, the SDH activity in the DOX group significantly decreased compared with that in the CON group, and the SDH activity significantly increased after 6‐SH intervention. The results indicated that 6‐SH significantly prevented DOX‐induced decreases in muscle mitochondrial levels in a dose‐dependent manner.

Skeletal muscle is composed of different types of muscle fibers, which are typically classified as slow‐twitch fibers, also known as type I fibers, and fast‐twitch fibers, also known as type II fibers, on the basis of their contraction speed. Muscle atrophy with different aetiologies often leads to different shifts in muscle fiber types. There are currently no reports on the type of muscle fiber shift that occurs during muscle atrophy induced by DOX. Compared with those in the CON group, the proportion of slow‐twitch fibers in the DOX group decreased, and the proportion of fast‐twitch fibers increased. This finding indicated that DOX induced a shift in muscle fibers from slow‐twitch fibers to fast‐twitch fibers, which is different from the shift that is caused by cancer cachexia. The 6‐SH intervention mitigated the effects of DOX in a dose‐dependent manner (Figure 4A,D).

To further investigate the changes in muscle fiber types and subsequent shift in fibers type ratio during the chronic toxicity of DOX, we performed immunofluorescence staining on gastrocnemius tissue. The results are shown in Figure 4B; fast‐twitch fibers were stained red and slow‐twitch fibers were stained green. Fluorescence quantitative analysis revealed that DOX led to synchronous atrophy of both slow‐twitch fibers and fast‐twitch fibers, and this effect was attenuated by treatment with 6‐SH (Figure 4E). Consistent with the results obtained through SDH staining, the proportion of slow‐twitch fibers decreased in the DOX group, indicating that DOX‐induced chronic toxicity is accompanied by a slow‐to‐fast fiber type shift. Importantly, 6‐SH interrupted the shift in skeletal muscle fiber type in a dose‐dependent manner.

### 3.4 6‐SH Attenuated DOX‐Induced Cardiotoxicity, Cardiac Fibrosis, and Cardiac Atrophy

**FIGURE 5:**
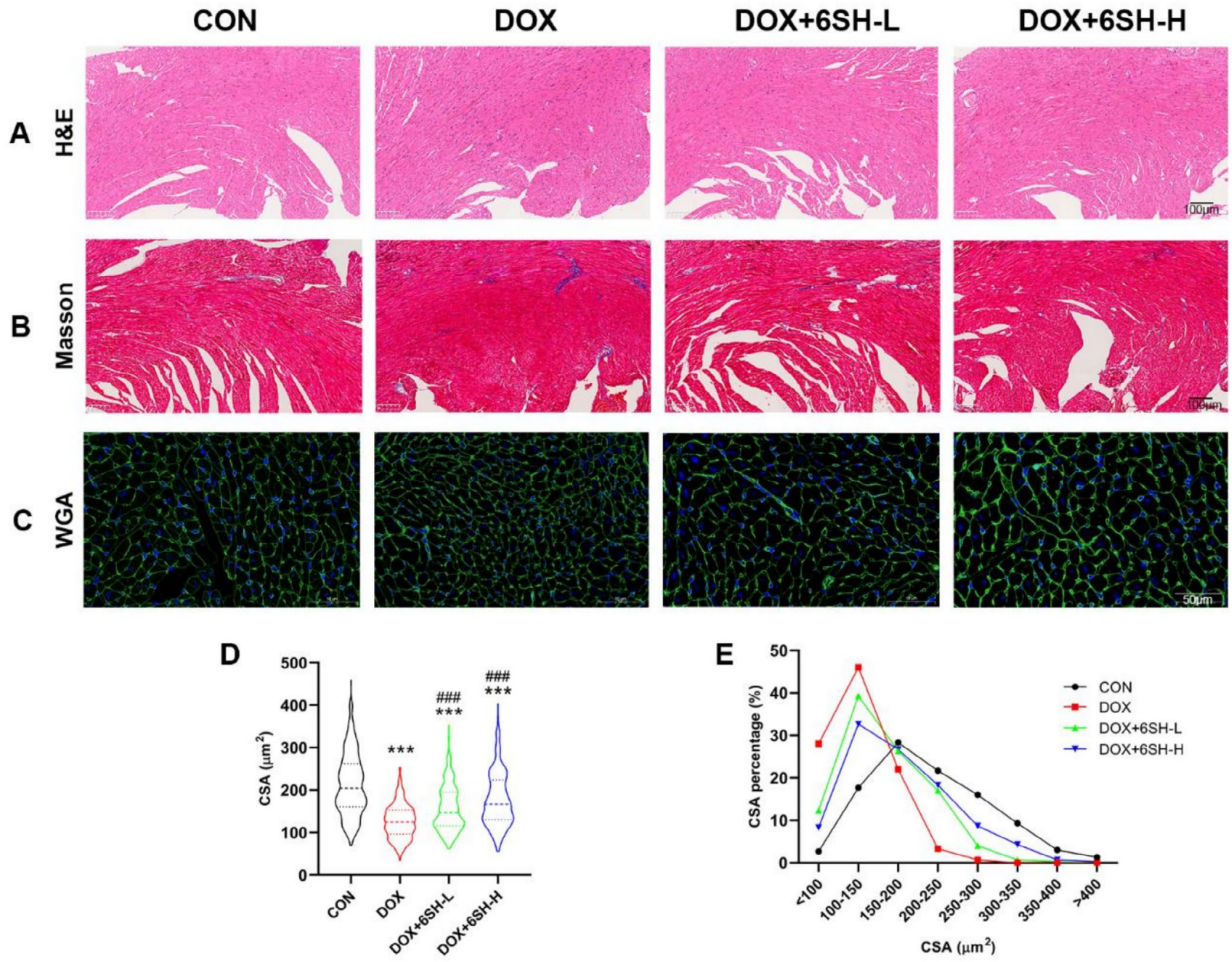
6‐SH alleviated cardiac injury, myocardial fibrosis and cardiac atrophy induced by DOX. (A) H&E‐stained sections of mouse hearts. (B) Masson‐stained sections of mouse hearts. (C) WGA‐stained sections of mouse hearts. (D) Statistical analysis of the CSA of the mouse heart (*n* = 300). (E) Distribution of the CSA of the mouse heart (*n* = 300). ***p < 0.001 versus the CON group; ###*p* < 0.001 versus the DOX group.

Studies have shown that a cumulative dose of DOX of 20 mg/kg can trigger cardiotoxicity, leading to pathological alterations in cardiac tissue (Li et al. 2025). The histological changes in the tissues were evaluated via H&E staining. As shown in Figure 5A, compared with those in the CON group, the hearts of the mice in the DOX group displayed disorganized myofibers, myocyte deformation, necrosis, inflammatory cell infiltration, and vacuolation. However, the mice in the DOX+6SH‐L and DOX+6SH‐H groups exhibited pathology indicative of mild cardiac damage, with preserved myofiber arrangement and reduced myocardial deformation and necrosis, along with minimal inflammatory cell infiltration.

The extent of myocardial fibrosis was evaluated via Masson's trichrome staining, as shown in Figure 5B. Compared with those in the CON group, the hearts of the mice in the DOX group presented significantly more myocardial fibrosis, whereas myocardial fibrosis was alleviated in the 6‐SH treatment groups.

Cardiac tissues were stained with WGA for CSA analysis of myocardial cells, as shown in Figure 5C,D. Compared with that in the CON group, the CSA of myocardial cells in the DOX group was significantly smaller. The distribution of the myocardial cell CSA curve shifted to the left of the *X*‐axis in the DOX group (Figure 5E). Importantly, the CSA in the DOX + 6SH‐L and DOX + 6SH‐H groups was significantly greater than that in the DOX group. Collectively, these results suggested that 6‐SH alleviated the DOX‐induced reduction in the myocardial CSA in a dose‐dependent manner.

### 3.5 6‐SH Mitigated the Decrease in Cardiac Contractile and Diastolic Functions Induced by DOX

**FIGURE 6:**
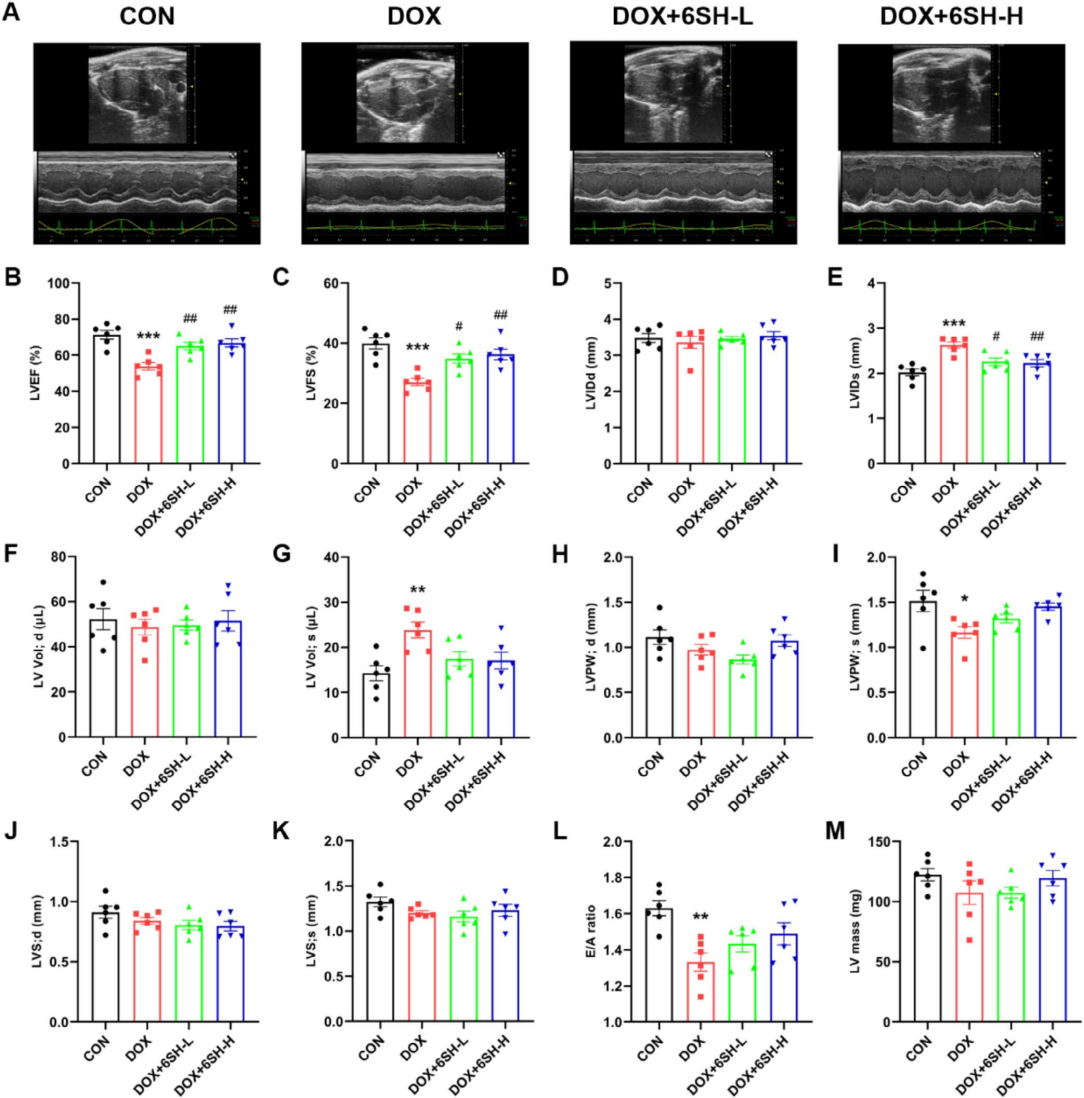
6‐SH alleviated the decrease in cardiac systolic and diastolic function induced by DOX. (A) Representative echocardiography images of mouse hearts. (B–M) Echocardiography parameters. (B) Left ventricle ejection fraction (LVEF). (C) Left ventricle fractional shortening (LVFS). (D) Left ventricle diastolic internal dimension (LVIDd). (E) Left ventricle systolic internal dimension (LVIDs). (F) Left ventricle diastolic volume (LVvol;d). (G) Left ventricle systolic volume (LVvol;s). (H) Left ventricle diastolic posterior wall thickness (LVPWd). (I) Left ventricle systolic posterior wall thickness (LVPWs). (J) Left ventricle diastolic interventricular septum thickness (LVS;d). (K) Left ventricle systolic interventricular septum thickness (LVS;s). (L) E/A ratio. (M) Left ventricle mass. The data are shown as the means ± SEM, *n* = 6/group, *p < 0.05, **p < 0.01, ***p < 0.01 versus the CON group; #*p* < 0.05, ##*p* < 0.01 versus the DOX group.

In this study, DOX‐induced chronic cardiotoxicity was further confirmed by echocardiographic assessments, which revealed decreased cardiac contractility and diastolic function relative to healthy samples. Echocardiographic images of the mouse hearts are depicted in Figure 6A. As shown in Figure 6B,C, LVEF and LVFS were significantly lower in the DOX group than in the CON group. Following intervention with 6‐SH, the LVEF and LVFS were significantly improved. The results indicated that DOX induced a significant decrease in cardiac contractile function, and this decrease was mitigated by 6‐SH. With respect to other cardiac functional parameters, DOX treatment did not significantly alter LVIDd or LVvol;d, but it led to a significant increase in the LVIDs and the LVvol;s and a significant decrease in the LVPWs. These alterations were reversed by the 6‐SH intervention. In terms of diastolic function, the E/A ratio significantly decreased in the DOX group but increased following 6‐SH intervention (Figure 6L). These findings suggest that DOX impairs cardiac diastolic function, but this impairment can be alleviated by 6‐SH intervention.

### 3.6 6‐SH Attenuated DOX‐Induced Oxidative Stress, Cardiac Injury, and Inflammation

**FIGURE 7:**
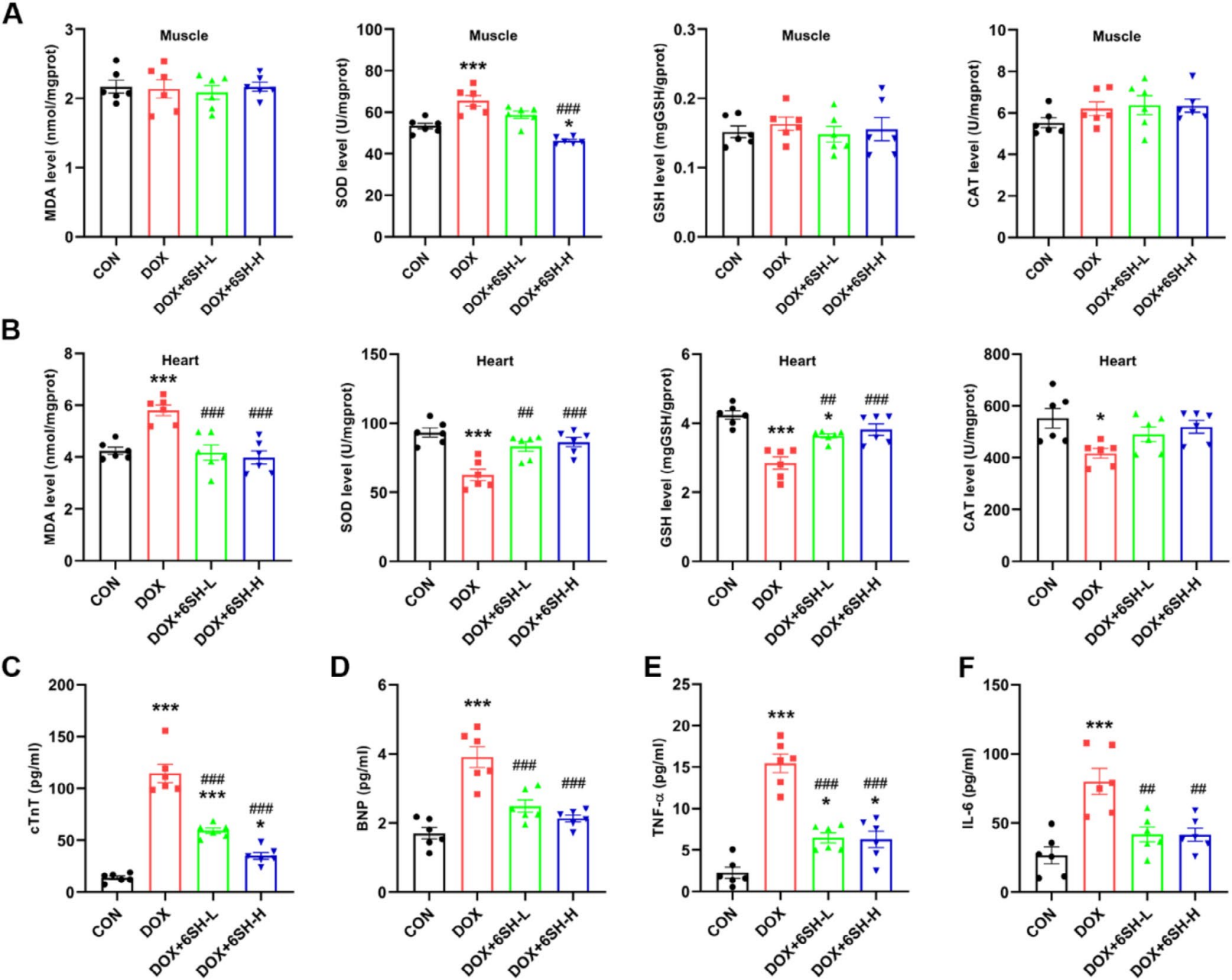
6‐SH attenuated DOX‐induced oxidative stress, cardiac injury, and inflammation. (A) The levels of MDA, SOD, GSH, and CAT in the gastrocnemius. (B) The levels of MDA, SOD, GSH, and CAT in the heart. (C) Serum cTnT levels. (D) Serum BNP levels. (E) Serum TNF‐α levels. (F) Serum IL‐6 levels. The data are shown as the means ± SEM, *n* = 6/group, *p < 0.05, ***p < 0.001 versus the CON group; ##*p* < 0.01, ###*p* < 0.001 versus the DOX group.

MDA, SOD, GSH and CAT levels in heart and muscle tissues were detected to evaluate DOX‐induced oxidative stress. DOX‐treated mice exhibited significantly increased oxidative stress in the heart, whereas no obvious changes were observed in the muscle (Figure 7A,B). In the gastrocnemius, the MDA, GSH and CAT levels were unchanged in the DOX group, but the SOD levels were increased compared with those in the CON group (Figure 7A). However, in the heart, the MDA level significantly increased in the DOX group, and the SOD, GSH and CAT levels significantly decreased. The administration of 6‐SH significantly attenuated DOX‐induced oxidative stress in the heart (Figure 7B).

We further investigated the cardiac injury biomarkers cTnT and BNP. DOX significantly increased cTnT and BNP levels compared with those in the CON group. The coadministration of 6‐SH with DOX resulted in a significant decrease in cTnT and BNP (Figure 7C,D). Moreover, DOX treatment was associated with increased levels of proinflammatory cytokines, as demonstrated by the TNF‐α and IL‐6 levels in the serum. 6‐SH exerted an anti‐inflammatory effect by suppressing TNF‐α and IL‐6 (Figure 7E,F).

### 3.7 6‐SH Regulated E3 Ubiquitin Ligases and Myogenic Factors in Skeletal Muscle and the Heart

**FIGURE 8:**
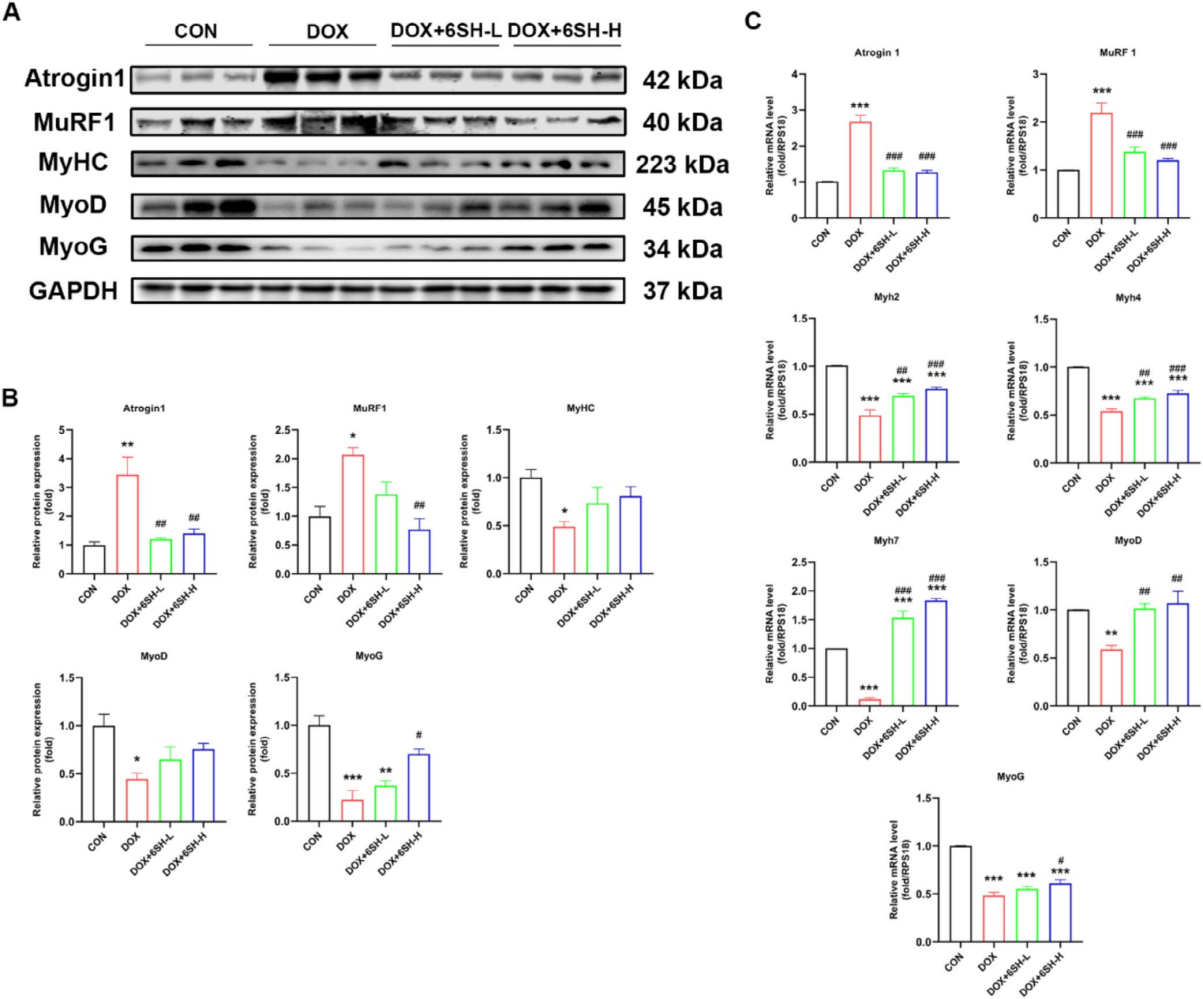
Effects of 6‐SH on E3 ubiquitin ligases and myogenic factors in the gastrocnemius after DOX treatment. (A) The protein expression of Atrogin1, MuRF1, MyHC, MyoD, and MyoG was evaluated via western blotting. GAPDH was used as an internal control. (B) The relative expression levels of the proteins were quantified via ImageJ software, normalized to the level of GAPDH, and normalized to the levels in the CON group (*n* = 3/group). (C) The mRNA expression of Atrogin1, MuRF1, Myh2, Myh4, Myh7, MyoD, and MyoG in gastrocnemius was assessed via qRT–PCR, and RPS18 was used as an internal control (*n* = 5/group). The data are presented as the means ± SEM, *p < 0.05, **p < 0.01, ***p < 0.001 versus the CON group; #*p* < 0.05, ##*p* < 0.01, ###*p* < 0.001 versus the DOX group.

Muscle atrophy is often associated with the activation of protein degradation pathways, which is usually accompanied by the activation of the E3 ubiquitin ligases Atrogin1 and MuRF1 and the downregulation of myogenic regulatory factors. Atrogin1 and MuRF1 are markers of skeletal atrophy. MyoD and MyoG are important myogenic regulatory factors that mediate muscle differentiation and they regulate MyHC expression. A reduction in their expression is thought to be an important pathological change associated with muscle atrophy. Our results demonstrate that DOX significantly upregulated the protein levels of Atrogin1 and MuRF1 and significantly downregulated MyHC, MyoD, and MyoG protein expression in the gastrocnemius (Figure 8A,B). After 6‐SH intervention, we observed dose‐dependent decreases in the expression of Atrogin1 and MuRF1 and increases in the expression of MyHC, MyoD, and MyoG. We further used qRT–PCR to evaluate mRNA expression in the gastrocnemius. Different muscle fiber types express different types of MyHC: MyHC I is encoded by Myh7, and MyHC II is encoded by Myh2 and Myh4. 6‐SH treatment increased the mRNA expression of Myh2, Myh4, and Myh7 in the gastrocnemius. Moreover, the Myh7 mRNA level increased significantly and by the greatest amount. No significant differences were observed in MyoG mRNA expression among the groups (Figure 8C).

**FIGURE 9:**
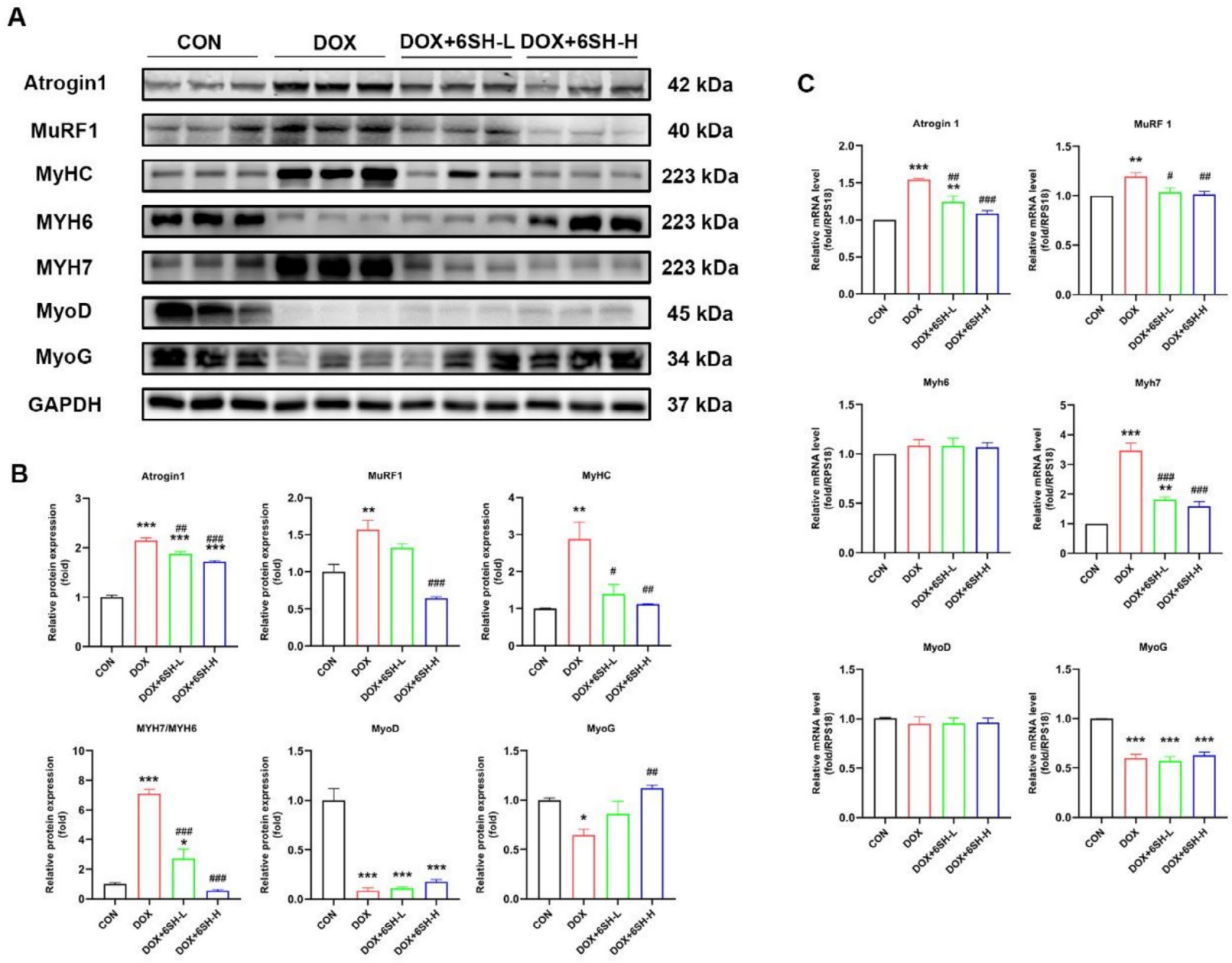
Effects of 6‐SH on E3 ubiquitin ligases and myogenic factors in the heart after DOX treatment. (A) The protein levels of Atrogin1, MuRF1, MyHC, MYH6, MYH7, MyoD and MyoG were evaluated via western blotting. GAPDH was used as an internal control. (B) The relative expression levels of the proteins were quantified via ImageJ software, normalized to the levels of GAPDH, and normalized to the levels in the CON group (*n* = 3/group). (C) The mRNA expression of Atrogin1, MuRF1, Myh6, Myh7, MyoD and MyoG in the heart was assessed via qRT–PCR, and RPS18 was used as an internal control (*n* = 5/group). The data are presented as the means ± SEM, *p < 0.05, **p < 0.01, ***p < 0.001 versus the CON group; #*p* < 0.05, ##*p* < 0.01, ###: *p* < 0.001 versus the DOX group.

DOX activates Atrogin1 or MuRF1 in cardiomyocytes both in vitro and in vivo, although the relevant findings remain inconsistent (Willis et al. 2019, 2009; Yamamoto et al. 2008). We further explored the effects of DOX and 6‐SH on E3 ubiquitin ligases and myogenic regulatory factors in the heart. As shown in Figure 9, DOX significantly increased the expression of Atrogin1 and MuRF1, and coadministration of 6‐SH with DOX reversed these phenomena. However, the effects of DOX on myogenic factors in the heart differ from those in skeletal muscle. DOX significantly decreased the protein expression of MyoD and MyoG but increased the protein expression of MyHC in the heart. The predominant MyHC isoform underwent characteristic atrophic switching, from MYH6 (α‐MYH) to MYH7 (β‐MYH). DOX increased the protein level of MYH7 and decreased the protein level of MYH6, leading to a significant increase in the MYH7/MYH6 ratio. 6‐SH decreased the protein level of MyHC and inhibited the switch from MYH6 to MYH7. 6‐SH treatment increased the protein expression of MyoG but did not increase the mRNA expression of MyoG in the heart. The protein and mRNA levels of MyoD in the heart were unchanged after 6‐SH treatment.

### 3.8 6‐SH Inhibited DOX‐Induced Necroptosis in Cardiac and Skeletal Muscle

**FIGURE 10:**
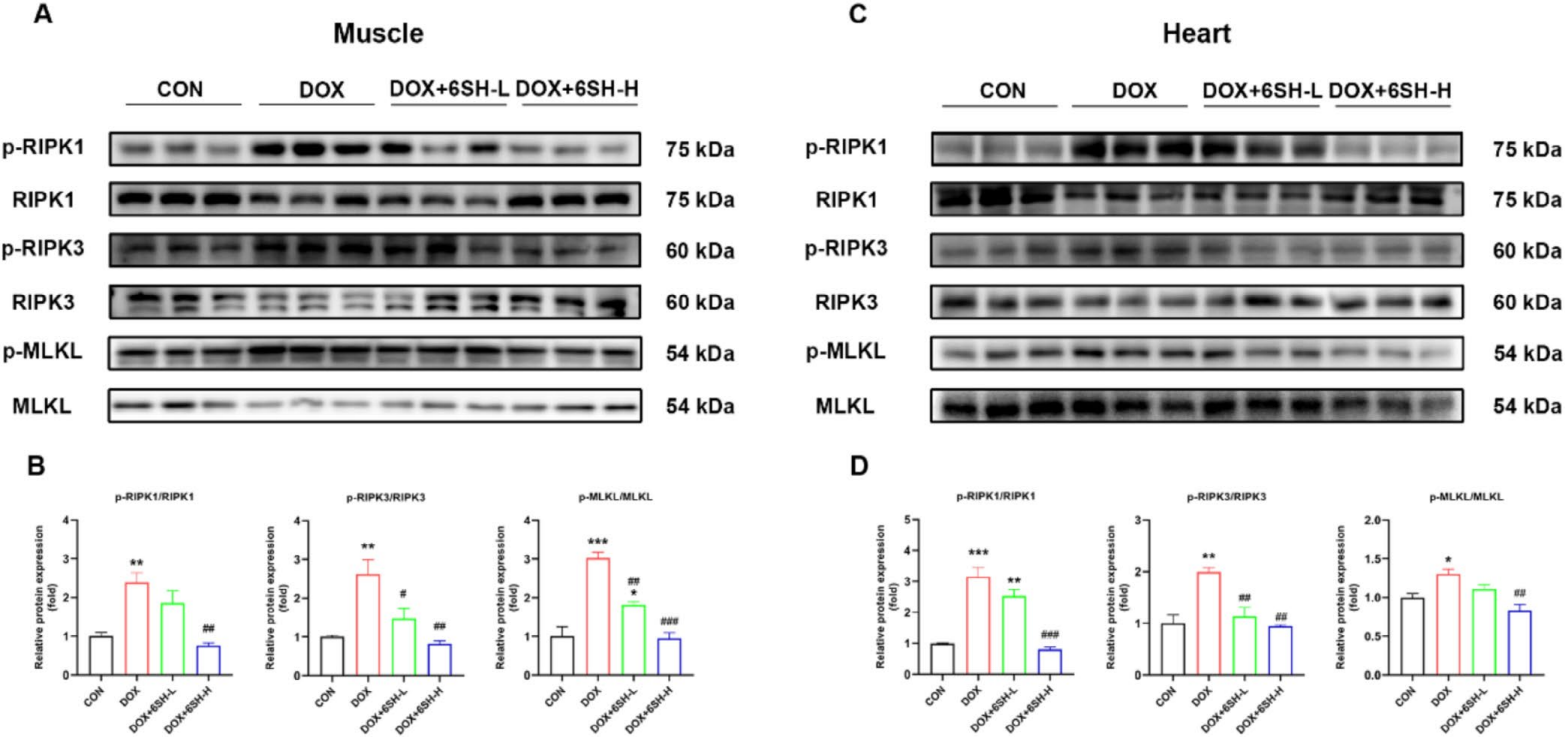
6‐SH inhibited DOX‐induced necroptosis in skeletal and cardiac muscle. (A) Representative blot bands of p‐RIPK1, RIPK1, p‐RIPK3, RIPK3, p‐MLKL and MLKL in the gastrocnemius. (B) Relative expression levels of necroptosis‐related proteins in the gastrocnemius. (*n* = 3/group). (C) Representative blot bands of p‐RIPK1, RIPK1, p‐RIPK3, RIPK3, p‐MLKL and MLKL in the heart. (D) Relative expression levels of necroptosis‐related proteins in the heart. (*n* = 3/group). The data are presented as the means ± SEM, *p < 0.05, **p < 0.01, ***p < 0.001 versus the CON group; #*p* < 0.05, ##*p* < 0.01, ###*p* < 0.001 versus the DOX group.

Numerous studies indicate that necrosis and apoptosis are central to anthracycline‐mediated cardiac injury. However, the role of necroptosis is still unclear. Here, we investigated the role of necroptosis in DOX‐induced cardiac and skeletal muscle atrophy, as shown in Figure 10. The levels of phosphorylated RIPK1 at serine 166 (p‐RIPK1^ser166^)/RIPK1, phosphorylated RIPK3 at Ter231 and Ser232 (p‐RIPK3^T231/S232^)/RIPK3, and phosphorylated MLKL at serine 358 (p‐MLKL^ser358^)/MLKL were significantly increased in muscle after DOX treatment and were reduced by cotreatment with 6‐SH (Figure 10A,B). Similarly, the protein levels of p‐RIP1^ser166^/RIPK1, p‐RIPK3^T231/S232^/RIPK3, and p‐MLKL^ser358^/MLKL were significantly increased in the heart. The DOX‐induced phosphorylation of RIPK1/RIPK3/MLKL was also attenuated following 6‐SH treatment (Figure 10C,D).

## 4 Discussion

Previous studies have shown that cardiac and skeletal muscle atrophy concomitantly develop with DOX‐induced cardiotoxicity (Hulmi et al. 2018; Montalvo et al. 2020). There is complex crosstalk between DOX‐induced cardiotoxicity, cardiac atrophy, and skeletal muscle atrophy. DOX can directly cause skeletal muscle atrophy, and its cardiotoxicity‐induced cardiac dysfunction further promotes skeletal muscle atrophy, which is a complication known as cardiac cachexia (Cella et al. 2024; Doerr et al. 2020; Kazemi‐Bajestani et al. 2014; Rausch et al. 2021). Furthermore, DOX directly causes cardiac atrophy, and cardiac atrophy can exacerbate the cardiotoxicity of DOX (Chen et al. 2022). Therefore, we propose that the alleviation of cardiac atrophy could protect against DOX‐induced cardiotoxicity. Although the mechanism of cancer cachexia‐induced skeletal muscle atrophy has been widely studied, the pathological mechanisms of DOX‐induced cardiac and skeletal muscle atrophy are not yet fully understood. Both cardiac and skeletal muscle are striated muscles. Notably, despite sharing numerous analogous physiological characteristics, they also exhibit distinct functional and structural differences. Owing to the lack of effective therapeutic options, elucidating the pathogenesis of DOX‐induced cardiac and skeletal muscle atrophy and developing a treatment strategy are crucial. This study demonstrated that 6‐SH treatment alleviated the skeletal muscle atrophy, cardiac atrophy, and cardiotoxicity induced by DOX. 6‐SH treatment of mice markedly increased body weight, skeletal muscle mass, and heart mass; attenuated the decrease in food intake; and ameliorated cachexia symptoms in a dose‐dependent manner. Moreover, 6‐SH did not compromise the antitumor activity of DOX; unexpectedly, it enhanced the antitumor efficacy of DOX. The mechanism of this synergistic enhancement remains unclear and merits further investigation. It may be attributed to the intrinsic antitumor activity of 6‐SH (Figueroa‐Gonzalez et al. 2024), as well as its capacity to modulate the accumulation and metabolism of antitumor agents in tumor cells (Mehanna et al. 2024).

Skeletal muscle atrophy can affect specific fiber types, slow‐twitch or fast‐twitch, and is often accompanied by a slow‐to‐fast or fast‐to‐slow fiber type shift. For example, muscle disuse leads to atrophy of slow‐twitch fibers, with a slow‐to‐fast fiber type shift, whereas cancer cachexia or fasting leads to preferential atrophy of fast‐twitch fibers, with a fast‐to‐slow fiber type shift (Ciciliot et al. 2013). Cachexia caused by heart failure or COPD leads to limb muscle atrophy with a slow‐to‐fast fiber type shift, but it leads to diaphragm atrophy with a fast‐to‐slow fiber type shift (Ciciliot et al. 2013). The mechanism by which DOX induces a muscle fiber type shift remains unclear. Our study confirmed that DOX mainly reduces type I muscle fibers, leading to a slow‐to‐fast fiber type shift. This shift is different from that observed in cancer cachexia. 6‐SH treatment interrupted the shift of skeletal muscle fiber types.

The myosin heavy chain (MyHC) is integral to muscle function, particularly in muscle contraction and interaction with actin (Lewis and Ochala 2023). Decreased MyHC expression is a significant indicator of muscle atrophy, as it reflects reduced muscle formation and differentiation. Our results revealed a significant decrease in the level of MyHC in skeletal muscle after DOX treatment, whereas DOX induced cardiac atrophy with a significant increase in MyHC protein levels. MyHC is essential for cardiac contraction and energy flow, and its dysregulation is closely linked to heart failure (LeWinter and Palmer 2015; Zhang et al. 2021). There are two MyHC isoforms: α‐MHC (fast, high ATPase activity) and β‐MHC (slow, low ATPase activity). In heart failure, there is a shift from α‐MHC to β‐MHC in the atrium, leading to reduced contractile function. This shift is associated with a decrease in myofibrillar protein production and diminished ATPase activity, contributing to weakened heart muscle (LeWinter and Palmer 2015). Therefore, we hypothesize that during DOX‐induced chronic cardiotoxicity pathogenesis, cardiac atrophy initially occurs, and it is followed by an increase in MyHC protein expression and a shift in MyHC isoforms, ultimately leading to pathological cardiac hypertrophy.

Studies suggest that elevated ROS formation plays an important role in DOX‐induced cardiac and skeletal muscle atrophy (Okutsu and Yamada 2023; Rausch et al. 2021; Rawat et al. 2021). DOX accumulates within the mitochondria of cardiac and skeletal muscle, resulting in the generation of ROS and ultimately mitochondrial dysfunction (Doerr et al. 2020; Wu et al. 2022). However, our study revealed that DOX caused different degrees of oxidative damage to cardiac and skeletal muscle tissue. DOX induced severe oxidative damage in the heart, which was evidenced by increased levels of the lipid peroxidation product MDA and decreased levels of the antioxidant substances SOD, CAT, and GSH. SDH staining results showed that DOX decreased skeletal muscle mitochondrial content significantly but did not cause oxidative damage; however, DOX treatment increased SOD levels. This difference indicates that ROS play different roles in DOX‐induced cardiac and skeletal muscle atrophy.

Muscle atrophy is caused by an increase in protein degradation coupled with a decrease in protein synthesis (Rausch et al. 2021). One efficient approach for preventing muscle atrophy is to suppress excessive protein degradation. The ubiquitin proteasome pathway, which involves E1, E2, and E3 enzymes, is one of the main proteolytic systems. Atrogin1 and MuRF1 are muscle‐specific E3 ubiquitin ligases that are significantly upregulated during muscle atrophy, so they are considered hallmarks of muscle atrophy. Knockout studies confirm their non‐exclusive yet critical role in muscle atrophy (Koyama et al. 2008; Willis et al. 2019; Yuan et al. 2015). DOX triggers severe muscle atrophy via the increases in oxidative stress and upregulation of the atrogenes Atrogin1 and MuRF1 in vivo and in vitro (Okutsu and Yamada 2023). Nevertheless, the effects of distinct chemotherapies on Atrogin1 and MuRF1 are inconsistent (Pedrosa et al. 2023). E3 ubiquitin ligases also have critical roles in the pathophysiology of cardiovascular diseases. Expression of these ligases is known to be correlated with myocardial infarction, heart failure, myocardial hypertrophy, fibrosis, and cardiac remodeling (Pagan et al. 2013). DOX upregulated Atrogin1 in heart and skeletal muscle through activation of the p38‐MAPK pathway and contributed to muscle atrophy in cardiac myocytes (Yamamoto et al. 2008). Willis et al. reported that dose‐related increases in the expression of MuRF1, but not Atrogin1, occurred in the heart. Both MuRF1 and Atrogin1 mRNA expression are significantly increased in the gastrocnemius after DOX treatment (Willis et al. 2019). MuRF1 knockout mice are protected from cardiac atrophy and exhibit no reduction in contractile function (Willis et al. 2019). The inhibition of MuRF1 by the small molecule Myomed#‐205 or the AKR1B1 inhibitor NARI‐29 markedly attenuated DOX‐induced cardiomyopathy, cardiac atrophy and heart mass loss (Alves et al. 2024; Syamprasad et al. 2023). Our results confirmed that Atrogin1 and MuRF1 play important roles in DOX‐induced cardiac and skeletal atrophy, but it is unclear whether their functions are independent.

MyoG and MyoD are two of the four myogenic regulatory factors that play distinct regulatory roles in muscle growth, development, and differentiation. MyoD participates in the formation and proliferation of myogenic cells, whereas MyoG regulates the terminal differentiation of myoblasts (Zammit 2017). Cancer cachexia or chemotherapy significantly inhibited MyoD and MyoG expression in muscle. Unlike muscle tissue, the myocardium has a limited capacity for regeneration, particularly in adult mammals (Weinberger and Riley 2024). Therefore, the role of myogenic regulatory factors in cardiac atrophy has not received abundant attention. Liu et al. demonstrated that MyoG was the target for DOX‐induced cardiotoxicity and, for the first time, revealed that DOX modulates the MyoG gene at the transcriptional, post‐transcriptional, translational, and post‐translational levels (Liu et al. 2015). DOX also inhibits the function of MyoD, and this impact is associated with myofibrillar loss in DOX‐induced cardiomyopathy (Kurabayashi et al. 1994). Our results revealed that DOX significantly reduced MyoD and MyoG levels in cardiac and skeletal muscle, but 6‐SH differentially regulated these genes. 6‐SH did not upregulate either the protein or mRNA levels of MyoD in the heart; these data indicate that 6‐SH may not affect the proliferation of myogenic cells but may stimulate only the terminal differentiation of myoblasts in the heart. The mechanism by which 6‐SH regulates myogenic factors remains unclear. E3 ubiquitin ligases, ROS, and inflammation are known to regulate myogenic factors, and 6‐SH has been shown to negatively regulate these.

The inflammatory cytokines IL‐6 and TNF‐α are linked to cachexia and muscle atrophy. They can activate E3 ubiquitin ligases or the NF‐κB pathway, leading to muscle atrophy and reduced muscle regeneration (Rausch et al. 2021; Wang et al. 2023). In mice with DOX‐induced cardiotoxicity, inflammatory responses are also exacerbated, leading to cardiac death and muscle atrophy (Dessouki et al. 2020; Khuanjing et al. 2021). Little is known about the function of inflammation in cardiac atrophy. 6‐SH has strong anti‐inflammatory activity and can inhibit leukocyte infiltration, reduce inflammatory mediators such as COX‐2 or iNOS, and affect NF‐κB and MAPK signaling (Bischoff‐Kont and Furst 2021). The results here show that 6‐SH reduced the levels of TNF‐α and IL‐6 in the serum of mice with DOX‐induced cardiotoxicity.

Proinflammatory cytokine activation during necroptosis is a canonical pathway. To date, relatively few studies have investigated the role of necroptosis in cancer‐ or chemotherapy‐induced cardiac and muscle atrophy. Recently, dexrazoxane and donepezil were shown to attenuate DOX‐induced inflammation and necroptosis (Khuanjing et al. 2021; Yu et al. 2020). These studies suggest a role for necroptosis in DOX‐induced cardiotoxicity. In terms of muscle atrophy, relevant findings concerning necroptosis are scarce. Muscle atrophy is often closely related to chronic inflammation. Given the relationship between the inflammatory response and necroptosis, necroptosis may be a contributing factor in the pathogenesis of muscle atrophy. Guo et al. demonstrated that necroptosis mediated muscle protein degradation in lipopolysaccharide‐induced cachexia (Guo et al. 2023). Other studies have shown that RIPK3‐mediated necroptosis is involved in muscular dystrophy (Bencze et al. 2023; Mariot et al. 2021). Inhibition of necroptosis improves both the histological features of muscles and the cardiac function of patients with Duchenne muscular dystrophy (DMD) (Bencze et al. 2023). Our results indicated that in cardiac and skeletal muscle subjected to chronic toxicity of DOX, the key components that mediate necroptosis, RIPK1, RIPK3, and MLKL, were all phosphorylated and activated. In agreement with our findings, 6‐SH also inhibited necroptosis in mouse models of acute kidney injury and nonalcoholic steatohepatitis (Gwon et al. 2021; Yang et al. 2024).

Our work still has certain limitations. This study demonstrates that E3 ubiquitin ligases, myogenic regulatory factors, and necroptosis play critical roles in DOX‐induced cardiotoxicity. However, the potential regulatory crosstalk among these three biological components remains to be fully elucidated, as does the precise molecular mechanism by which DOX and 6‐SH modulate the activity of E3 ubiquitin ligases. In subsequent research, we will employ gene knockout or overexpression of MuRF1 or Atrogin1 to investigate the alterations in myogenic regulatory factors and necroptosis, thereby clarifying whether a regulatory crosstalk exists between them.

## 5 Conclusion

Cardiac and skeletal muscle atrophy concomitantly develop with DOX‐induced chronic cardiotoxicity. 6‐SH negatively regulated E3 ubiquitin ligases, necroptosis, MyHC isoform shift, oxidative stress, and inflammation, while upregulating myogenic regulatory factors, thereby alleviating DOX‐induced cardiac atrophy, skeletal muscle atrophy, and cardiotoxicity.

## Author Contributions

**Xipeng Sun:** funding acquisition, investigation, methodology, project administration, resources, writing – original draft, writing – review and editing. **Yaxian Wang:** methodology, project administration, validation. **Quanjun Yang:** data curation, supervision. **Bo Xin:** methodology, supervision, validation. **Jinlu Huang:** data curation, software, supervision. **Cheng Guo:** conceptualization, funding acquisition, supervision, writing – review and editing.

## Funding

This work was supported by the National Natural Science Foundation of China, 81803633, 82274151.

## Conflicts of Interest

The authors declare no conflicts of interest.

## Data Availability

The data that support the findings of this study are available from the corresponding author upon reasonable request.
